# Spanish Translation and Psychometric Validation of a Measure of Acculturative Stress among Latinx Immigrants in the USA

**DOI:** 10.3390/ijerph19052808

**Published:** 2022-02-28

**Authors:** Kritzia Merced, Chimdindu Ohayagha, Ria Grover, Isis Garcia-Rodriguez, Oswaldo Moreno, Paul B. Perrin

**Affiliations:** 1 Hunter Holmes McGuire VA Medical Center, Central Virginia Veterans Affairs Health Care System, Richmond, VA 23249, USA; kritzianel.merced-morales@va.gov; 2 Department of Psychology, Virginia Commonwealth University, Richmond, VA 23284, USA; ohayaghac@mymail.vcu.edu (C.O.); groverr@vcu.edu (R.G.); garciaroi@vcu.edu (I.G.-R.); oamoreno@vcu.edu (O.M.)

**Keywords:** acculturative stress, discrimination, immigration, Latinx, Hispanic, Spanish

## Abstract

Background: In the United States, the Latinx community is growing at a faster rate than any other racial or ethnic minority group. Members of this community have been found to experience a number of acculturative stressors after immigrating, including xenophobia, racism, and discrimination. Although several scales have been created in recent years to measure acculturative stress in Spanish-speaking immigrants, they are long, do not have nuanced subscales, or have not been validated in an extremely diverse sample of Latinx immigrants. Objective: The purpose of the current study was to translate and psychometrically validate the Riverside Acculturative Stress Inventory (RASI) in a diverse sample of Spanish-speaking immigrants. Methods: A sample of 202 Latinx immigrants in the United States completed the RASI as well as measures of depression and anxiety. Results: An initial confirmatory factor analysis suggested that the overall subscale factor structure was not an ideal fit for the data. An exploratory factor analysis suggested the retention of four subscales, each with three items, forming a 12-item Spanish RASI short form. As indices of convergent validity, the RASI total score was positively associated with depression and anxiety. Conclusions: The findings from the study contribute to the literature a brief and valid assessment of acculturative stress in Spanish-speaking immigrants. The RASI Spanish short form holds promise to stimulate research on the unique adversities experienced by Latinx immigrants.

## 1. Introduction

Over the past few decades, immigration has been a prominent contributing factor to population growth in the United States. Public opinion regarding immigration is often a point of contention, and recent historical events regarding the immigration of Spanish-speaking groups have exacerbated the American political divide [1]. The population of racial/ethnic minority groups has been steadily increasing [2], with the most growth occurring in Spanish-speaking and Latinx populations, which now makes up the largest racial/ethnic minority and immigrant group [3]. In 2018, the Spanish-speaking population accounted for approximately 18% (60 million) of the U.S. population [4]. Reports indicate that 37 million Latinx individuals (34.8%) ages 5 and older speak Spanish at home, making Spanish the most common non-English language in the United States [4,5]. It has been projected that Spanish-speaking and Latinx individuals will make up 27% of the nation’s population by 2060 [6].

The initial influx of Latinx immigrants to the United States has largely been influenced by the abolishment of the Immigration Act of 1965, removing the national quota system, which limited the immigration of Spanish-speaking groups [2]. Although Latinx groups make up the largest immigrant population, immigration from Latin American countries to the United States has been on the decline since the 2000s [7]. In 2000, approximately 40% of all Latinx individuals in the United States were foreign-born as compared to 33% in 2017; immigration from Mexico, which has been noted historically as the largest source of U.S. immigrants, has dramatically declined since 1965 [8].

Given that Latinxs account for a significant portion of the U.S. population, which informs public health agendas, it is important to identify factors that may impact their physical and psychological health, as well as overall well-being [3]). Although the United States has been referred to as the land of opportunity, the immigration process often entails various challenges. Acculturation into a country involves learning aspects of a new culture, including learning a new language, value system, and norms [9,10]. These changes can bring several stressors that directly affect one’s mental and physical health [3]. Stressors, as well as the response to certain conditions that happen before, during, and after immigration, all cumulatively combine to inform the construct that is known as acculturative stress [3].

Many stressors arise during the immigration process. These stressors can be broken down into structural and social levels [7]. Structural influences include immigration policies dictating access to health care, education, housing, transportation, and employment [7,11,12,13,14]. Immigrant tensions intensified due to the Mexican border wall initiative as well as due to the withdrawal of the protection of the Deferred Action for Childhood Arrivals (DACA) program [15]. The fear of being persecuted by immigration officials is a larger systemic structural stressor that impacts Latinx communities. In a small qualitative study by Pinedo et al. [7] examining immigration-related stressors, Latinx individuals often expressed intense feelings of persecution or deportation by immigration officials, constantly feeling as if they were “on guard”. Additional stressors included discrimination, anti-immigration sentiments, negative media portrayals, stigma, and many others [7].

Both structural and social stressors can lead to physical and mental health disparities among Latinx communities. For example, depressive symptoms and high blood pressure have been linked to the effects of discrimination [3,16,17,18]. In the same study by Pinedo and colleagues [7], 77% of Latinx participants reported recognizing anti-immigration sentiments in their area, 41% reported seeing or experiencing an immigration raid, and 82% personally knew someone who had been deported [7]. Additionally, 64% of participants had symptoms of anxiety, 50% had symptoms of depression, 64% had symptoms of a drug use disorder, and 40% had symptoms of an alcohol use disorder [7]. High acculturative stress has been directly related to mental health issues in Latinx Americans [10]. Relative to mental health, little is known regarding the physical health consequences that stem from acculturative stress [3]. HIV risk, intimate partner violence, and substance abuse have all been shown to be negatively associated with acculturative stress [3]. Constant exposure to stress can lead to harmful coping strategies, giving rise to diseases such as heart disease and obesity [3,19]. Acculturative stress has negative outcomes in the context of blood pressure, asthma, sleep, body mass index, and overall physical health [3].

To date, there are limited measures that adequately assess acculturative stress. The most notable measures include the 60- and 17-item versions of the Academic, Familial, and Environmental Acculturative Stress Scale [20,21], the 22-item Migration–Acculturative Stressor Scale [22], the 36-item Acculturative Stress Scale for International Students [23], and the Acculturative Stress Questionnaire [24]. These measures assess multidimensional aspects of acculturative stress, such as perceived hate, enculturation pressures, stress due to cultural changes, guilt, social isolation, language competency pressures, unfair climate, and other acculturation-related constructs. Although these measures have demonstrated acceptable psychometric properties, many have a combination of limitations. Some were created for specific immigrant populations, which may be appropriate given that acculturative stress experiences can present differently among cultural groups, but it might inadvertently limit the utility of the measures in future research with culturally diverse populations. For example, the Academic, Familial, and Environmental Acculturative Stress Scale was normed for Korean American and Korean immigrant adolescents, and the Acculturative Stress Scale for International Students was normed for undergraduate international college students. The 22-item Migration–Acculturative Stressor Scale [22] showed good psychometric properties but within U.S. Chinese populations, and the Acculturation Stress Questionnaire was normed for Asian-American communities.

Currently, only three known measures assess acculturative stress in Spanish-speaking/Latinx communities—the Multidimensional Acculturative Stress Inventory for Adults of Mexican Origin (MASI) [25], the Acculturative Stress Inventory for children (ASIC) [26], and the Hispanic Stress Inventory version 1 and 2 (HSI, HSI-2) [27,28]. The MASI is a 36-item stress measure that assesses four acculturative stress-related factors salient for Mexican adults, including Spanish competency pressures, English competency pressures, pressures to acculturate, and pressures against acculturation [25]. The ASIC is an 8-item measure that assesses perceived discrimination and immigration-related experiences such as acculturative stress-related factors. The Hispanic Stress Inventory is a 59-item (United States-born version) and 73-item (immigrant version) measure that assesses the psychosocial stress among people of Latin-American origin. With these measures being the current acculturative stress measures for the Latinx population, they present several notable limitations regarding applicability to broader Latinx communities in the United States. The ASIC only measures two aspects of acculturation, which may limit the range of subconstructs measured, and the MASI and both versions of the HIS may be too long for practical administration in various communities and clinical settings.

By contrast, the Riverside Acculturation Stress Inventory (RASI) is a 15-item measure with five subscales developed by Benet-Martinez and Haritatos [29] to provide a brief but comprehensive measure of interpersonal, intellectual, professional, and structural pressures associated with acculturative stress [20,30]. The RASI has a variety of advantages that make it a robust yet nuanced measure of acculturative stress. It focuses on various aspects of acculturative stress, not solely relying on challenges with second culture or culture-of-origin issues like previous acculturative stress measures. Having a broader focus aligns with acculturation theory [31], denoting that stress can come from experiences with either culture. The RASI is also a brief measure, which may ameliorate participant burden and facilitate higher participant completion rates. The RASI evaluates culture-related challenges in five domains—language skills (e.g., misunderstanding due to one’s accent), work challenges (e.g., working harder than nonimmigrant/minority peers), intercultural relations (e.g., disagreements with others for behaving in ways that are “too American” or “too ethnic”), discrimination (e.g., being mistreated because of one’s ethnicity), and cultural/ethnic makeup of the community (e.g., living in an environment that is not culturally diverse) [29,32]. The internal consistency alphas for the Language Skills, Discrimination, Intercultural Relations, Cultural Isolation, and Work Challenges subscales were 0.84, 0.80, 0.75, 0.68, and 0.68, respectively [29]. Although there is evidence that the RASI is a reliable and valid measure for acculturative stress, participants in the original validation study were first-generation Chinese-American undergraduate students and older members of the university community. Some researchers also question the 5-factor structure of the model, given that the original study was an exploratory factor analysis with varimax rotation and a limited sample size [32]. 

To date, there is no existing measure of acculturative stress in Spanish for the U.S. Latinx population that is both parsimonious to reduce participant burden yet nuanced enough to capture the multidimensional aspects of acculturative stress. As a result, the current study aimed to translate the RASI into Spanish and psychometrically validate it in a diverse sample of Spanish-speaking immigrants.

## 2. Method

### 2.1. Procedure

This study was approved by the institutional review board at Virginia Commonwealth University (HM20000872), and the procedure was conducted in accordance with the Declaration of Helsinki. Participants were recruited as part of a convenience sample in a southeastern urban city and surrounding suburbs. The sample was recruited in-person from churches, restaurants, barber shops, primary care clinics, social service organizations, and sports associations, among other similar community organizations. Participants who met the inclusion criteria after being queried by one of two Spanish-speaking, Latina immigrant research assistants were provided the informed consent form for the survey, which they signed. To be included in this study, participants must have been (a) born in Latin America (including Puerto Rico and Brazil), (b) age 18 or older, and (c) able to read and write in Spanish via self-report. After the eligibility criteria were met and the consent form was signed, the participants were asked to fill out the questionnaires and demographic information, and they were paid USD 5 in cash. Data for this article were from a larger study on Latinx immigrant mental and physical health [33].

### 2.2. Participants

The participants were prescreened to ensure eligibility and then provided an informed consent form in Spanish. Of the initial 207 participants who completed the study, data from five participants were removed from the database due to greater than 50% missingness on the RASI. The final sample size was N = 202. The average age of the 202 participants was 36.61 (*SD* = 12.50); most identified as women (63.9%), married (48%), and working full-time (37.1%). In terms of family gross income in the past year, most participants indicated an income of less than $15,000 (41.6%), followed by participants indicating an income between $15,000 and $35,000 (25.7%). Regarding the highest education level achieved, most participants reported having a high school diploma (38.1%), followed by some college experience (16.8%), or having a college diploma (15.4%). The mean age at which participants had immigrated to the United States was 22.32 (*SD* = 10.28). The greatest number of participants came from Mexico (28.2%), although there was tremendous diversity in terms of country of origin. See Table 1 for the complete demographic information.

### 2.3. Measures

The Riverside Acculturation Stress Inventory (RASI) is a 15-item scale that measures 5 domains of acculturative stress, including intercultural relations, language skills, discrimination, work challenges, and cultural/ethnic makeup of the community [34]. Each item is rated on a 5-point scale ranging from 1 (strongly disagree) to 5 (strongly agree). In the RASI, higher scores reflect higher levels of acculturative stress, ranging from 15 to 75 points. The measure was originally developed to provide a brief but comprehensive measure of the main acculturative stressors affecting Chinese-Americans living in the United States [33]. Accordingly, for the purpose of this study, the RASI was adapted and translated from English to Spanish using Carter and Chapman’s [35] method. This involved translation from English to Spanish by a bicultural and bilingual researcher, followed by back-translation by another bicultural and bilingual researcher. Any discrepancies between the versions were mutually resolved through discussions by the research team.

The Generalized Anxiety Disorder Scale (GAD-7) [36] is a 7-item self-report scale that measures anxiety symptoms. Total scores range from 0 to 21, and higher scores reflect higher anxiety symptoms. For the current study, the GAD-7 showed good internal reliability (α = 0.92).

The Patient Health Questionnaire (PHQ-9) [37] is a 9-item self-report scale that measures depression symptoms. Total scores range from 0 to 27, and higher scores reflect higher depression symptoms. The PHQ-9 showed good internal reliability in the current sample (α = 0.86).

### 2.4. Data Analyses

#### Primary Analyses

For the participants who had 50% missing items or fewer on the RASI, the expectation maximization algorithm was used to impute the other missing item values from the items that were present (as noted above, five participants were removed for having greater than 50% missingness). Item-level missingness was extremely low, with only 1–3 missing data points per item, with the exception of Item 6, which had 5 missing data points. In order to examine whether the theorized 5-factor structure (Benet-Martinez, 2003) fit for the Spanish version of the RASI, a confirmatory factor analysis (CFA) was run using all 15 items. To evaluate the fit of the model, the normed fit index (NFI ≥ 0.95), relative fit index (RFI ≥ 0.95), incremental fit index (IFI ≥ 0.90), Tucker–Lewis Index (TLI ≥ 0.90), comparative fit index (CFI ≥ 0.95), and root mean square error of approximation (RMSEA ≤ 0.10) were calculated and evaluated according to Meyers and colleagues’ cutoff suggestions [38]. A follow-up exploratory factor analysis (EFA) with principal axis factoring and a promax rotation was performed to determine whether a different factor structure might be operating in the data. The Cronbach’s αs, means, and standard deviations were calculated. Finally, for convergent validity, correlations among the RASI, GAD-7, and PHQ-9 were calculated.

## 3. Results

**Figure 1:**
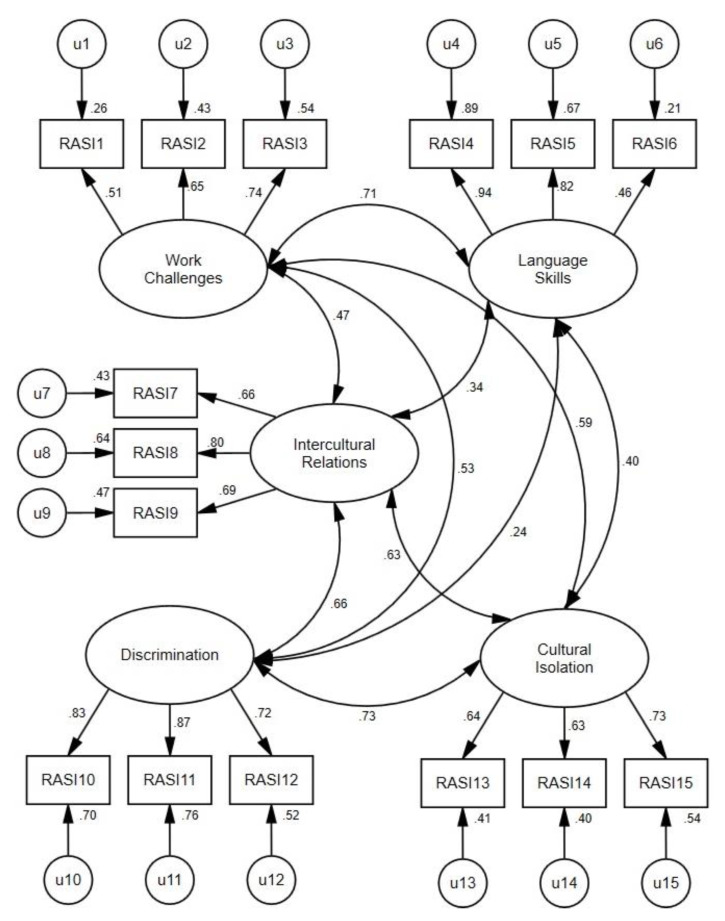
5-factor CFA for the 15-item Riverside Acculturation Stress Inventory in Spanish. Note: All item loadings are standardized loadings.

The 5-factor CFA of the 15 items from the RASI suggested poor fit across the fit indexes except for the IFI, CFI, and RMSEA, which were in the acceptable range (Table 2). All item loadings onto their latent construct were statistically significant (all *p*s < 0.001) and greater in magnitude than 0.46 (Figure 1). The correlation coefficients among all five latent factors ranged between *r* = 0.24 and *r* = 0.73, suggesting that the proposed domains were distinct but related components of a broader acculturative stress construct.

**Figure 2:**
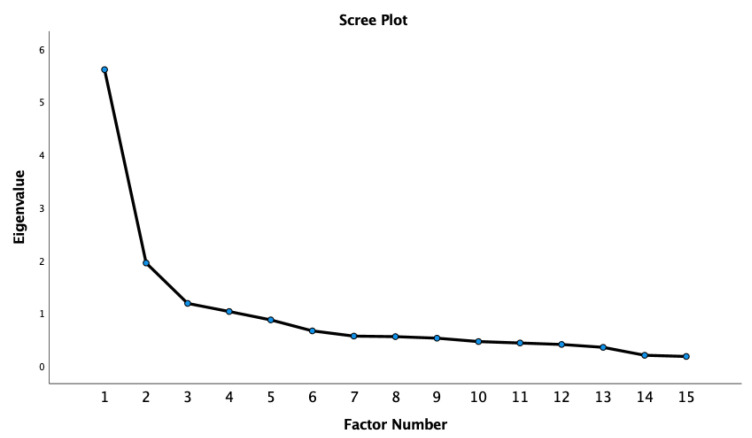
EFA Scree plot.

Due to the mostly poor fit indexes, an exploratory factor analysis (EFA) was run to determine whether a better factor solution might be operating among the 15 items of the Spanish version of the RASI. The EFA yielded four factors with eigenvalues greater than 1 (Figure 2). The first factor with an eigenvalue of 5.60 accounted for 37.38% of the total variance in the data. The second factor had an eigenvalue of 1.95 and accounted for an additional 12.99%. The third factor had an eigenvalue of 1.18 and accounted for an additional 7.89%. The fourth factor had an eigenvalue of 1.03 and accounted for an additional 6.87%. Tachnick and Fidell [39] argue, a factor solution should account for at least 50% of the item variance. The first two accounted for 50.37%, the first three for 58.26%, and the first four for 65.13%, suggesting that between 2–4 factors should be retained. Further, the Scree plot revealed a pronounced inflection point at the third eigenvalue, suggesting the possible retention of three factors, pending an analysis of simple structure.

Three items achieved simple structure (defined as a loading of 0.40 or greater in magnitude on the primary factor and no secondary loading within a 0.15 magnitude of the primary loading) for factor 1, three items for factor 2, three items for factor 3, and three items for factor 4 (Table 3). Three factors retained all original items from the proposed subscales in the 5-factor CFA model—the Intercultural Relations, Discrimination, and Cultural Isolation factors. The fourth factor included items from the initial Work Challenges and Language Skills subscales theorized by the scale creators [29,32]. Three items that were originally included in the Work Challenges and Language Skills subscales did not map on in any discernible way to the original subscales. Due to this item-loading pattern, Items 1, 2, and 6 were eliminated in favor of retaining four factors in the Spanish version of the RASI.

A Cronbach’s α for the 12-item-version (α = 0.82) suggested adequate internal consistency for the full scale. The Cronbach’s αs for the four subscales were α = 0.81 for the first factor (Work and Language Challenges), α = 0.84 for the second factor (Discrimination), α = 0.75 for the third factor (Intercultural Relations), and α = 0.71 for the fourth factor (Cultural Isolation), further suggesting good and acceptable ranges. All correlations among the subscales were statistically significant, as were the total scores of the PHQ-9 and GAD-7, showing evidence of the RASI subscales’ convergent validity (Table 4). The correlation between the 15-item and 12-item version was *r* = 0.98, suggesting near-complete singularity in the overall acculturative stress construct being measured by each version, and again pointing toward the superiority of the short form for this population based on the principle of parsimony. The Spanish version of the 12-item RASI short form appears in Appendix A.

## 4. Discussion

The purpose of this study was to translate and psychometrically validate the Riverside Acculturative Stress Inventory (RASI) in a diverse sample of 202 Spanish-speaking immigrants in the United States. The sample completed the RASI as well as measures of depression and anxiety. An initial confirmatory factor analysis suggested that the overall subscale factor structure was not an ideal fit to the data. Exploratory factor analysis (EFA) suggested the retention of four subscales, each with three items, forming a 12-item Spanish RASI short form. As indices of convergent validity, the RASI total score was positively associated with depression and anxiety.

The results generally show that the RASI Spanish short form had similar psychometric properties to the original slightly longer version validated with Chinese immigrants [29] and was highly correlated with it, though the original scale needed some tweaking in order to increase its utility in Spanish and with Latinx immigrants in the United States. The final four-factor structure suggested the multi-dimensional nature of acculturative stress in Latinx immigrants involves work and language challenges, discrimination, intercultural relations, and cultural isolation. However, three of the original items from the RASI (e.g., having to work harder than most Americans, feeling pressure to represent other Hispanics well, and being bothered by having an accent) did not map on well to the original Work Challenges and Language Skills subscales. Further, the EFA suggested that these two sub-constructs would be better combined into one overall work and language challenges construct. This set of findings may be due in part to the unique acculturative stress experiences encountered by the current community sample of Spanish-speaking adults in the southeastern region of the United States. Immigration trends suggest that the southeastern United States is becoming a growing area of Latinx immigrants, particularly from Central American countries [40,41]. The southeastern United States also provides diverse employment opportunities in agriculture, factory, and manual labor that immigrants largely occupy [41]. As a result, in the current sample, work and language challenges may have been so intertwined that they form a single sub-component of acculturative stress, and many of the language challenges could in fact have been occurring in a work environment.

The four RASI short form subscales each had acceptable internal consistency and were moderately correlated with each other (Table 4), supporting the notion that the RASI short form subscales overlap in the context of a larger acculturative stress construct but also measure coherent and different aspects of acculturative stress. The short form total was associated with both depression and anxiety, providing evidence of convergent validity and suggesting acculturative stress may impact mental health and overall well-being, supporting the study of Latinx immigrants through a biopsychosocial lens [42]. These findings are also consistent with previous research on minority stressors and mental health among the U.S. Latinx population [43,44]. 

The current findings shed light on the importance of capturing multidimensional aspects of acculturative stress in a brief format. For decades, many measures that incorporated multidimensional domains entailed longer and time-consuming scales that evoked participant burden. Participant burden is important to consider for Latinx immigrant communities since they already tend to underutilize healthcare services [45,46,47] or are more hesitant to participate in research [48,49]. If they do participate, they tend to terminate early when compared to other non-Latinx communities [50]. Having short and validated measures, like the RASI Spanish short form, may reduce burden, which in turn may promote healthcare utilization, foster equitable and represented scholarship, and reduce overall Latinx disparities.

Given that the current findings shed light on the associations between acculturative stress and mental health outcomes such as depression and anxiety, it is imperative for researchers to also examine or control for mental health when including acculturative stressors in their research. Relatedly, it is important for clinicians to explore dimensions of depression and anxiety when Latinx immigrants are presenting with acculturative stress in clinical practice. In doing so, researchers and clinicians will gain knowledge and awareness and be able to apply culturally sensitive skills when working with these communities. By engaging in deeper knowledge, awareness, attitudes, and skills surrounding Latinxs’ acculturative stressors, researchers and clinicians can further engage in cultural humility. Most importantly, using this validated RASI Spanish short form will also allow for researchers and clinicians to engage in more culturally sensitive means by examining and exploring the lived experiences of Latinx immigrants in their native and/or preferred language.

In terms of study strengths, the RASI short form was validated in this study in a diverse community sample of Latinx immigrants, transcending the limitations of many previous measures validated by using convenience sampling from colleges and universities (e.g., Acculturative Stress Scale for International Students) [23]. In particular, the use of snowball and community sampling, as in the current study, often helps access difficult-to-reach participants, including those who are not historically represented in the psychological and sociological scientific literature (e.g., BIPOC, low-income, immigrant) [51]. Relatedly, another strength of the study is the diversity in the countries of origin in the sample. This diversity allows for a broader representation in the constellation of the acculturative experiences of Latinx immigrants in the United States.

## 5. Limitations and Future Directions

While this study validated a novel measure of acculturative stress in Spanish-speaking immigrants, there are some notable limitations that should be considered and, as a result, future directions for research. First, the sample consisted of a large proportion of women, consistent with many other studies in psychological science. Future studies should attempt to recruit a more equal gender balance in order to help the current findings generalize more strongly to men. Second, participants’ education levels varied dramatically, ranging from post-graduate to high school education and below. This facilitated greater community representation by the study sample, but it may have inadvertently masked participants with reading and comprehension difficulties. Given the potential variability in reading and comprehension levels, future studies should ensure that administration of the RASI is sensitive to diverse education and reading levels by providing both written and oral administration options. Third, although there was great diversity in terms of the countries of origin of the participants, the sample only came from the southeastern region of the United States. Given the cultural variability in the United States, future research should study acculturative stress in other regions or sub-regions. Fourth, although the sample size was robust enough for the current analyses, larger samples may help further elucidate the functionality of the RASI short form. Fifth, it would be important to test the reliability of the RASI short form across varying Spanish-speaking populations from different countries, even more so than the extremely diverse sample recruited in the current study. Lastly, future studies should consider an exploratory approach and assess how cultural differences within Spanish-speaking communities may contribute to a diversity of acculturative stress conceptualizations. Overcoming these limitations may help further validate the Spanish RASI short form and better measure acculturative stress in Latinx immigrants in the United States.

## 6. Conclusions

The purpose of the current study was to translate and psychometrically validate the RASI in a diverse sample of Spanish-speaking immigrants. The results of the study suggest the retention of four subscales, each with three items, forming a 12-item Spanish RASI short form. More broadly, these findings contribute to the literature a brief and valid assessment of acculturative stress in Spanish-speaking immigrants. Overall, the RASI Spanish short form holds promise to stimulate research on the unique adversities experienced by Latinx immigrants.

## Figures and Tables

**Table 1:** Participant characteristics.

Variable	Category	Numerical Value	
Age, M *(SD)*		36.61 (12.50)	
Sex, *n* (%)	Male	69 (34.2)	
	Female	129 (63.9)	
Marital Status (%)	Single	63 (31.2)	
	Married	97 (48.0)	
	Divorced	11 (5.4)	
	Separated	5 (2.5)	
	Widowed	1 (0.5)	
	Open Union	18 (8.9)	
	Other	4 (2.0)	
Education, *n* (%)	<High School	44 (21.8)	
	High School	77 (38.1)	
	Some College	34 (16.8)	
	College Graduate	31 (15.4)	
	Post-Graduate	11 (5.4)	
Current Employment Status, *n* (%)	Full-Time	75 (37.1)	
	Part-Time	30 (14.9)	
	Homemaker	39 (19.3)	
	Unemployed	21 (10.4)	
	Student	2 (1.0)	
	Hourly Pay Work	13 (6.4)	
	Volunteer Work	4 (2.0)	
	Retired	8 (4.0)	
Family Gross Income (%)	<$15,000	84 (41.6)	
	$15,000–$35,000	52 (25.7)	
	$35,001–$55,000	28 (13.9)	
	$55,001–$75,000	11 (5.4)	
	>$75,000	9 (4.5)	
Health Insurance Status (%)	Yes	63 (31.2)	
	No	139 (68.8)	
Access to Medical Care (%)	Yes	120 (59.4)	
	No	82 (40.6)	
Country of Origin (%)	Mexican	57 (28.2)	
	Salvadoran	40 (19.8)	
	Guatemalan	33 (16.3)	
	Honduran	19 (9.4)	
	Dominican	11 (5.4)	
	Puerto Rican	13 (6.4)	
	Peruvian	7 (3.5)	
	Colombian	6 (3.0)	
	Cuban	3 (1.5)	
	Bolivian	3 (1.5)	
	Nicaraguan	3 (1.5)	
	Venezuelan	2 (1.0)	
	Paraguayan	1 (.5)	
	Argentinian	1 (.5)	
	Brazilian	1 (.5)	
	Other	2 (1.0)	
Age immigrated to the U.S., M *(SD)*		22.32 (10.28)	
Another family member had already immigrated to the U.S. (%)	First person to immigrate	51 (25.2)	
	Not first person to immigrate	146 (72.3)	

Note: Not all values add up to 202 participants because of missing data.

**Table 2:** Fit indices for the RASI 5-factor CFA.

Fit Index	5-Factor (15 Items)
NFI	0.86
RFI	0.80
IFI	0.92
TLI	0.88
CFI	0.92
RMSEA	0.07

Note: NFI = normed fit index; RFI = relative fit index; IFI = incremental fit index; TLI = Tucker–Lewis index; RMSEA = root mean square error of approximation.

**Table 3:** EFA item loadings for 15-item the Riverside Acculturation Stress Inventory.

Item	Factor			
	1	2	3	4
RASI4 ^WL^	**0.96**	−0.13	0.06	−0.08
RASI5 ^WL^	**0.79**	−0.12	0.03	0.01
RASI3 ^WL^	**0.62**	0.26	−0.09	−0.02
RASI2 ^r^	0.45	0.41	−0.06	−0.10 ^r^
RASI1 ^r^	0.37	0.30	−0.15	0.03 ^r^
RASI11 ^D^	−0.03	**0.91**	0.02	−0.06
RASI10 ^D^	0.00	**0.64**	0.18	0.02
RASI12 ^D^	−0.01	**0.50**	0.13	0.24
RASI7 ^IR^	−0.12	0.05	**0.72**	−0.03
RASI8 ^IR^	0.00	0.13	**0.71**	−0.05
RASI9 ^IR^	0.07	0.04	**0.69**	−0.05
RASI6 ^r^	0.29	−0.17	0.37	0.24 ^r^
RASI13 ^CI^	−0.10	−0.05	−0.07	**0.88**
RASI15 ^CI^	−0.03	0.27	0.04	**0.50**
RASI14 ^CI^	0.31	0.04	−0.02	**0.47**

Note: Extraction Method: Principal axis factoring. Rotation Method: Promax with Kaiser normalization. Bolded values reflect items achieving simple structure loading on the respective factor. ^r^ reflects items that were removed because they did not achieve simple structure. ^WL^ = Work and Language Challenges; ^D^ = Discrimination; ^IR^ = Intercultural Relations; and ^CI^ = Cultural Isolation.

**Table 4:** Convergent validity.

Variable	1	2	3	4	5	6
1. RASI Short Form Total						
2. RASI Work and Language Challenges	-					
3. RASI Discrimination	-	0.292 **				
4. RASI Intercultural Relations	-	0.292 **	0.559 **			
5. RASI Cultural Isolation	-	0.383 **	0.584 **	0.455 **		
6. Depression	0.161 *	0.015	0.118	0.191 *	0.176 *	
7. Anxiety	0.184 *	0.083	0.135	0.220 **	0.117	0.638 **

Note: ** Correlation was significant at the 0.01 level. * Correlation was significant at the 0.05 level.

## Data Availability

Data are available from the corresponding author upon request.

## Appendix A

**Table A1:** RASI Spanish short form.

Muy en Desacuerdo (1)-Muy de Acuerdo (5)
1. ^WL^ Cuando ando en busca de trabajo, a veces siento que mi origen hispano es una limitación. [In looking for a job, I sometimes feel that my Hispanic background is a limitation.]
2. ^WL^ Es difícil para mi desempeñar bien mi trabajo debido a mi inglés. [It’s hard for me to perform well at work because of my English skills.]
3. ^WL^ Frecuentemente me siento incomprendido o limitado en situaciones cotidianas debido a mi inglés. [I often feel misunderstood or limited in daily situations because of my English skills.]
4. ^IR^ He tenido desacuerdos con otros hispanos (amigos o familia) por gustarme costumbres Americanas o por mi manera de hacer cosas. [I have had disagreements with other Hispanics (e.g., friends or family) for liking American customs or ways of doing things.]
5. ^IR^ He tenido desacuerdos con Americanos por gustarme las costumbres hispanas o mi manera de hacer cosas. [I have had disagreements with Americans for liking Hispanic customs or ways of doing things.]
6. ^IR^ Siento que mis costumbres (Hispanas o Americanas) han causado conflicto en mis relaciones. [I feel that my particular practices (Hispanic or American) have caused conflict in my relationships.]
7. ^D^ He sido tratado groseramente o injustamente debido a mi origen hispano. [I have been treated rudely or unfairly because of my Hispanic background.]
8. ^D^ Me he sentido discriminado por Americanos debido a mi origen hispano. [I have felt discriminated against by Americans because of my Hispanic background.]
9. ^D^ Siento con frecuencia que gente interpreta mi comportamiento basado en sus estereotipos en como son los hispanos.
10. ^CI^ Siento que no hay suficiente gente hispana en mi entorno. [I feel that there are not enough Hispanic people in my living environment.]
11. ^CI^ Cuando estoy en un lugar o cuarto donde soy la única persona hispana a menudo me siento diferente o aislado. [When I am in a place or room where I am the only Hispanic person, I often feel different or isolated.]
12. ^CI^ Siento que el ambiente donde vivo no es suficiente multicultural; no tiene suficiente riqueza cultural. [I feel that the environment where I live is not multicultural enough; it does not have enough cultural richness.]

Note: ^WL^ = Work and Language Challenges; ^D^ = Discrimination; ^IR^ = Intercultural Relations; and ^CI^ = Cultural Isolation.
